# Analysis of Non-responders to LNG-IUS in Cases of Heavy Menstrual Bleeding

**DOI:** 10.1007/s13224-025-02141-5

**Published:** 2025-09-23

**Authors:** Gayatri Savani, Saroj Kumari, Shruti Ravinarayan, Nigamananda Mishra

**Affiliations:** 1 Department of Obstetrics and Gynaecology, Bhabha Atomic Research Centre Hospital, Mumbai, Maharashtra India; 2 Department of Obstetrics and Gynaecology at Pt, Madan Mohan Malviya Shatabdi Hospital, Govandi, Mumbai, Maharashtra India

**Keywords:** Heavy menstrual bleeding (HMB), Levonorgestrel-intrauterine system (LNG-IUS), Non-responders

## Abstract

**Introduction:**

Heavy menstrual bleeding (HMB) or menorrhagia, a common complaint among women during their reproductive years, significantly impacts women's quality of life, productivity, and healthcare costs. The treatment options range from conservative medical management to minimally invasive procedures and hysterectomy. While levonorgestrel-intrauterine system (LNG-IUS) is established as an effective treatment for HMB, there is a lack of significant research on factors determining non-response in women with HMB. This study aims to analyse the factors behind non-response to LNG-IUS in women with HMB.

**Material and Methods:**

A retrospective observational study was conducted at BARC Hospital, Mumbai, from January 2017 to March 2020, including 88 patients with HMB who received LNG-IUS. Non-responders to LNG-IUS were analysed for reasons of failure, including endometrial thickness, hysteroscopy, and biopsy reports. Patients were contacted one year later to assess treatment outcomes, and alternative treatments or surgery was offered to non-responders, with final histopathological findings documented.

**Results and Discussion:**

In this study, 29.5% of patients were non-responders, with the majority aged 40–44 years, and most experiencing persistent heavy bleeding, dysmenorrhea, device expulsion, or misplacement. Half of the non-responders had an endometrial thickness of less than 10 mm and proliferative endometrium on biopsy. Nine patients underwent hysterectomy, with adenomyosis found in seven of them.

**Conclusion:**

LNG-IUS is effective in treating HMB, but factors, such as age, endometrial thickness, and certain underlying structural and non-structural pathologies, may contribute to non-response.

## Introduction and Background

Menstruation is a physiological process associated with several disorders in which heavy menstrual bleeding is common [1]. Heavy menstrual bleeding can be defined as menstrual blood loss that is greater than 80 ml per cycle [2]. HMB can be measured objectively as well as subjectively. The current and most common definition of heavy menstrual bleeding is menstrual blood loss so excessive it interferes with a woman's physical, social, and emotional health and quality of life (QoL) [3].

The structural causes of HMB include polyp, adenomyosis, leiomyoma, and malignancy which can be evaluated by imaging or histopathology, while other non-structural causes include coagulopathy, ovulatory dysfunction, endometrial disorders like hyperplasia, iatrogenic causes, and not-yet-classified causes. The provider needs to choose the most likely etiology to effectively and appropriately manage these patients. The treatment varies from conservative medical management to minimally invasive procedures to hysterectomy.

The conservative management includes hormonal treatment such as medroxyprogesterone acetate, norethisterone acetate, and levonorgestrel-releasing intrauterine system (LNG-IUS). A systematic review and meta-analysis by Jin Qui et al. [4] revealed that the LNG-IUS was more effective for the management of menorrhagia as compared with conventional medical treatment.

A study by Nelson AL et al. revealed that several studies exist that have assessed LNG-IUS as a suitable alternative to hysterectomy in women with HMB. LNG-IUS has been approved in 120 countries worldwide for contraception and in 115 countries for the management of menorrhagia [5]. Another study by Georgy Joy Eralil showed that as compared to usual medical therapies for menorrhagia, LNG-IUS leads to greater improvement in heavy menstrual bleeding in women concerning their daily routine, as well as physical and mental well-being [6].

Though LNG-IUS devices resulted in a large reduction in menstrual blood loss, not all patients responded to this method of treatment. Lack of effectiveness (37%) and irregular or prolonged bleeding (28%), were cited as common reasons for discontinuation of the LNG-IUS, according to a study conducted by Middleton LJ and others [7].

While LNG-IUS has been established as a suitable modality of treatment for heavy menstrual bleeding, there is limited research on the factors determining non-response to LNG-IUS in women with HMB. Ultrasound, hysteroscopy, and histopathological findings in women with HMB who received alternate oral medical management and who underwent hysterectomy following non-response to LNG-IUS have not been previously studied. By conducting this study, we aim to analyse the factors responsible for non-response to LNG-IUS.

## Material and Methods

This retrospective observational study was conducted at BARC Hospital, Mumbai, on 88 patients with HMB who received LNG-IUS from January 2017 to March 2020. Patients suspected of malignancy or with contraindications to progesterone therapy were excluded. Patients were followed up one year post-study to assess treatment outcomes. Non-responders to LNG-IUS were analysed for reasons of treatment failure through their symptoms, measurement of endometrial thickness on ultrasound pre-insertion, hysteroscopy findings, and histopathological reports. Non-responders were offered alternative medical treatments as well as surgical options. Those who opted for surgical management underwent hysterectomy, and the final histopathological findings were documented. The findings from all the non-responders were subsequently analysed and evaluated.

### Statistical Methods

Qualitative variables were presented with numbers and percentages, while quantitative variables were presented with means and standard deviations. Data were analysed using the independent samples t test for quantitative variables and the χ^2^ test for qualitative variables. Statistical significance was set at *p* < 0.05.

## Results and Discussion

In our study, LNG-IUS was inserted in 88 patients with HMB, of which 26 (i.e. 29.5%) were non-responders. Among non-responders, 50% were aged 40–44 years, 30% were aged 45–49 years, 15.3% were aged 35–39 years, and 7.7% were aged 50–54 years. A majority (88.5%) of non-responders were multiparous. Most non-responders had multiple comorbidities (77%).

In a similar study conducted by Gupta Taru et al. [8], LNG-IUS was inserted in 70 patients with HMB with 8 non-responders (11.4%) (Table 1).

**Table 1:** Reasons for removal among non-responders

Reason for removal across non-responders	Dysmenorrhea	Misplaced LNG	Persistent HMB	Recurrent inflammation	Spontaneous expulsion	Spotting	Total
No. of patients	5	2	13	1	4	1	26

The primary reasons for LNG-IUS removal among non-responders included persistent HMB (50%), dysmenorrhea (19.23%), spontaneous expulsion (15.4%), and device misplacement (7.7%).

In a study by Pleun Beelen et al. [9], LNG-IUS was inserted in 201 patients, out of which 46% of women discontinued LNG-IUS. The most common reason is being persistent HMB (42%), f/b dysmenorrhea, and pelvic pain in 17%.

Gupta Taru et al. [8] in their study showed 30% of non-responders to have irregular bleeding, followed by 20% who had dysmenorrhea, 4.2% who had spontaneous expulsion, and 6.1% who had vaginitis (Table 2).

**Table 2:** Pre-LNG-endometrial thickness on USG (mm)

ET (mm)	Non-responders	Responders	Total	
< 10	13	33	46	0.446
10–15	11	21	32	
15–20	0	5	5	
20–25	2	2	4	
25–30	0	1	1	

Ultrasound findings before insertion showed that 50% of non-responders had an endometrial thickness of less than 10 mm, while 42.3% had ET of 10–15 mm and 7.7% had ET of 20–25 mm. No literature was found that correlated the success or failure of LNG-IUS with pre-insertion endometrial thickness (Table 3).

**Table 3:** Histopathological findings of endometrial biopsy

Histopathological findings	Non-responders	Responders	Total	
Proliferative	17	42	59	0.844
Secretory	4	7	11	
Other (disordered proliferative)	5	13	18	

Histopathological findings of endometrial biopsy revealed that the majority of patients with HMB had proliferative endometrium. Even among non-responders, 65.4% had proliferative endometrium, followed by 19.2% having disordered proliferative endometrium and 15.4% having secretory endometrium (Table 4).

**Table 4:** Hysteroscopy findings

Appearance of endometrium	Moderate	Profuse	Scanty	Total
Total	13	09	04	26

Another study conducted by Das Subrata et al. [10] also showed the majority of patients with HMB to have proliferative endometrium (41/150).

Gupta Taru et al. [8] in their study showed the majority of patients with HMB to have proliferative endometrium followed by disordered proliferative and lastly by secretory endometrium (Table 5).

**Table 5:** Reasons for removal versus treatment offered

	Medical	Surgical	Total
Dysmenorrhea	3	2	5
Misplaced LNG	2	0	2
Persistent HMB	9	4	13
Recurrent inflammation	1		1
Spontaneous expulsion	1	3	4
Spotting	1		1
Total	17	9	26

In our study, 50% of non-responders showed evidence of moderate endometrium on hysteroscopy, while 34.6% had profuse endometrium and 15.4% had scanty endometrium.

Out of the 26 non-responders in our study, 65.4% were managed with alternative medical management, most of which belonged to those with complaints of persistent HMB, while the remaining 34.6% were managed surgically (hysterectomy).

Pleun Beelen et al. [9], in their study, found that 32% of the patients who discontinued LNG-IUS were given additional medical management, while 14% of the non-responders underwent hysterectomy (Table 6).

**Table 6:** Surgical finding after hysterectomy

Post-op HPR	Adenomyosis	Adenomyosis with leiomyoma	Endometrial polyp	Leiomyoma	Total
No. of patients	4	3	1	1	9

Histopathological reports of those patients who went through surgical management showed 44.4% of patients to have adenomyosis, f/b 33.3% with adenomyosis and leiomyoma, and 11.1% each having endometrial polyp and leiomyoma, respectively.

A study by Eshna Gupta et al. [11] concluded that LNG-IUS is an effective method in the management of adenomyosis. Another study by Shuyi Chen et al. [12] also showed that LNG-IUS is effective in the management of types 1 and 2 of adenomyosis. However, it is seen from our study that although LNG-IUS is an effective method for the management of adenomyosis, the majority of the non-responders who underwent hysterectomy were found to have adenomyosis.

## Conclusion

The study demonstrates that while LNG-IUS is effective for the treatment of HMB, a significant proportion of patients do not respond to this treatment. Factors such as age, endometrial thickness, and certain underlying structural and non-structural pathologies play a role in non-response. Further research with a prospective study on a larger scale is needed to better understand these factors and improve treatment strategies for non-responders.
